# Tuning Surface Properties via Plasma Treatments for the Improved Capture of MicroRNA Biomarkers

**DOI:** 10.3390/ma15072641

**Published:** 2022-04-03

**Authors:** Giorgio Speranza, Gaetano Roberto Mele, Pietro Favia, Cecilia Pederzolli, Cristina Potrich

**Affiliations:** 1 Center for Sensors and Devices, Fondazione Bruno Kessler, Via Sommarive 18, 38123 Trento, Italy; speranza@fbk.eu (G.S.); megabob@hotmail.it (G.R.M.); pederzo@fbk.eu (C.P.); 2 Department of Industrial Engineering, University of Trento, v. Sommarive 9, 38123 Trento, Italy; 3 CNR-Istituto di Fotonica e Nanotecnologie, Via alla Cascata 56/C, 38123 Trento, Italy; 4 Department of Chemistry, CNR Inst. NANOTEC, University of Bari Aldo Moro, 70124 Bari, Italy; pietro.favia@uniba.it; 5 CNR-Istituto di Biofisica, Via alla Cascata 56/C, 38123 Trento, Italy

**Keywords:** plasma treatments, biofunctional surfaces, microRNAs, biomarker capture

## Abstract

Advanced materials could bring about fundamental improvements in the evolution of innovative analytical devices, i.e., biosensors or lab-on-a-chip devices, in particular in the context of liquid biopsies. Here, plasma deposition processes were tested for the introduction of primary amines on silicon surfaces by tuning the amounts and availability of amino-charged residues. Different binary (CH_4_/NH_3_) and ternary (CH_4_/NH_3_/H_2_ and CH_4_/NH_3_/N_2_) mixtures of gases were used as feeds for the plasma treatments. The obtained surfaces were fully characterized for their chemical and physical properties before their use as capture materials in a functional test. Synthetic and fluorescently conjugated microRNA-21 (miR-21) was selected as the target molecule. The capture of miR-21 increased linearly with the increase in amino nitrogen measured on surfaces. The surface showing the most promising performance was further analyzed in different conditions, i.e., varying pH and time of incubation, incubation with different microRNAs, and possible elution of captured microRNAs. The apparent pH range of primary amines present on the surfaces was around 3.5–4. Positively charged surfaces prepared via PE-CVD were, therefore, demonstrated as being suitable materials for the capture of microRNA biomarkers, paving the way for their inclusion in biomedical devices for the purification and analysis of circulating biomarkers.

## 1. Introduction

In recent years, the need for the minimally invasive and early diagnosis of severe pathologies has become even more urgent. Precision medicine meets this need by customizing health care to individual patients through new tools to classify and characterize diseases and their hosts. Among these new tools, liquid biopsies have the potential to improve the management of severe pathologies, such as neurodegenerative diseases, inflammatory syndromes, and tumor diseases, at different levels ranging from screening to monitoring, treatment response, and determination of the development of resistance in cancer [1,2,3]. Liquid biopsy approaches include the analysis of circulating tumor cells, circulating tumor DNA, circulating microRNAs, and tumor-derived extracellular vesicles, but are not limited to cancer diagnosis and monitoring. Several biomarkers or panels of biomarkers are, therefore, included in a liquid biopsy. Among biomarkers, microRNAs are emerging as promising analytes with high sensitivity and specificity [3,4]. MicroRNAs are a class of endogenous, non-coding, single-stranded RNA molecules measuring approximately 18–22 nucleotides, which emerged as key players in the post-transcriptional regulation of gene expression [4,5]. This class of RNAs has gained clinical relevance as their aberrant expression has been shown to correlate with the pathogenesis and progression of several diseases, including cancer [6,7,8]. Among them, miR-21 is one of most studied ‘oncomiRs’, whose overexpression has been detected in a variety of tumors [7,9,10,11]. Beside cancer, miR-21 is dysregulated in cardiovascular diseases [12,13], diabetes [14], and obesity [15]. In our research, miR-21 has been selected for a case study for the capture of circulating biomarkers on positively charged surfaces. MicroRNAs, as with all nucleic acids, are negatively charged molecules, meaning they are prone to being adsorbed on positively charged surfaces, as previously demonstrated [16]. The electrostatic forces are indeed the leading factors in promoting microRNA capture. Amino-terminated surfaces have been widely demonstrated as ideal substrates for adsorbing and purifying nucleic acids in a functional conformation [16,17,18,19]. In all cases, primary amino groups are grafted on silicon or polymeric materials via silanization with amino silane molecules. Another technique that is very effective in introducing amino groups onto surfaces relies on the functionalization processes in non-equilibrium (cold) plasmas [20,21]. Cold plasmas, namely ionized gases in non-equilibrium conditions, can be ignited at low (LP, tens–hundreds of Pa) and at atmospheric pressure (AP), and can modify the surfaces of materials by means of PE-CVD (plasma-enhanced chemical vapor deposition), etching (ablation), or grafting processes [22].

Several surface modification methods have been developed to create layers able to promote desirable interactions with biological materials while discouraging unwanted interactions. Most of these methods were first developed for materials devoted to biomedical applications, in particular for implants aimed at replacing or restoring the function of compromised or degenerated tissues or organs [23,24,25,26]. Among all techniques utilized for engineering surfaces, those based on plasma technology have been proven to be extremely efficient in defining surface properties simply by varying the plasma parameters [27,28,29,30,31]. In this work, plasma processes have been explored as an alternative method with respect to amino silanes for introducing amino groups on silicon surfaces with the aim of capturing microRNA biomarkers. Several binary and ternary mixtures of gas feeds were investigated for the plasma treatment, and the resulting surfaces were fully characterized from a chemical perspective via X-ray photoelectron spectroscopy (XPS) and fluorescamine assay. The performances of such surfaces were tested with a functional assay. Surfaces prepared with ternary mixtures of CH_4_/NH_3_/N_2_ gases were the most promising for possible implementation in microdevices for biomarkers analyses.

## 2. Materials and Methods

### 2.1. Materials

Monocrystalline silicon wafer diced in 1 cm × 1 cm substrates (FBK Microfabrication Facility) and fluorescamine (4′-phenylspiro (2-benzofuran-3,2′-furano)-1,3′-dione) were purchased from Thermo Scientific (Waltham, MA, United States). Trichloroethylene, acetone, and all powders for buffer solutions were purchased from Sigma-Aldrich s.r.l. (Milan, Italy). Synthetic hsa-miR-21 conjugated at the 5′ end with the fluorescent dye Alexa-488 (5′-Alexa488-UAGCUUAUCAGACUGAUGUUGA-3′; miR − 21) and synthetic hsa-miR-16 conjugated at the 5′ with the same dye (5′-UAGCAGCACGUAAAUAUUGGCG-3′) were synthetized by Integrated DNA Technologies (IDT, Leuven, Belgium).

### 2.2. Plasma Treatments

Silicon substrates were sonicated in trichloroethylene for 5 min before being plasma-coated. The plasma reactor consisted of a vacuum chamber maintained at 10^−7^ mbar during non-working conditions to avoid contamination. The plasma was ignited by a COPRA DN 200 CF generator (CCR GmbH, Troisdorf—Germany) operating at 13.56 MHz and generating a high-density plasma (>10^12^/cm^3^) with a magnetic field resonating at 2.45 GHz. The pressure was kept constant at 0.015 mbar for all processes, with the treatment time kept at 15 min and the power at 200 W, except in experiments where the power was varied to find the best experimental conditions.

Different binary (CH_4_/NH_3_) and ternary (CH_4_/NH_3_/H_2_ and CH_4_/NH_3_/N_2_) mixtures of gases were used as feeds, as described in Table 1. The total flow rate was kept constant at 80 sccm.

### 2.3. Characterizations

The chemical composition of the surfaces treated in different plasma conditions was analyzed by X-ray photoelectron spectroscopy (XPS). XPS analyses were performed using a Kratos Axis Ultra DLD (Kratos, Manchester, UK) instrument equipped with a hemispherical analyzer and a monochromatic Al Kα(1486.6 eV) X-ray source in spectroscopy mode. The emission angle between the axis of the analyzer and the normal to the sample surface was 0°. For each sample, a wide spectrum (pass energy at 160 eV) and the Si 2p, O 1s, C 1s, and N 1s high resolution core lines (pass energy 20 eV) were recorded. The N 1s core line was best fitted in two components (~400.5 and ~399 eV [32,33,34]), named here as amino and non-amino to account for the surface density of amino groups added to the surface with the plasma treatments. Charge compensation was performed and the CH_x_ component of the C 1s signal was set to 285 eV as the binding energy (BE) reference. Spectral analysis was performed using a software developed in house based on R libraries [35,36]. Core lines analysis was performed using a linear background subtraction and Gaussian components for peak fitting. The sensitivity factors provided by the instrument manufacturer were utilized for elemental quantification. BE values were assigned according to reference databases [37,38] and the literature [32,33,39].

A fluorescamine assay was employed to estimate the presence of primary amines on the treated surfaces, as they react to form a fluorophore exclusively in the presence of accessible amino groups [40]; the yield of fluorescent products was employed to assess the presence and availability of the amines on the surface. Fluorescamine powder was suspended in acetone to a working concentration of 1 mM and rapidly diluted four times in sodium phosphate buffer at 0.2 M, pH 8, directly on the surface. A 20 μL drop of 0.25 mM solution was incubated on the surface for 5 min in the dark. After incubation, the surfaces were inspected under a Leica DMLA fluorescence microscope (Leica Microsystems, Wetzlar, Germany) equipped with a Hg lamp and fluorescence filter cube A (Leica Microsystems, GER; 20× magnification objective; 303.8 ms exposure time). At least three images were recorded for each surface. Fluorescence analysis was performed with the Fiji software [41].

### 2.4. Functional Assays

A functional assay based on miRNA adsorption was selected to assess and compare the functional properties of the different plasma-functionalized surfaces [19,42]. Briefly, 10 ng of synthetic microRNA-21 (hsa-miR-21-Alexa488; miR-21) was dissolved in water and incubated on the plasma-treated surfaces for 20 min at room temperature, in the dark, washed with ultrapure water, and imaged using a Leica DMLA fluorescence microscope equipped with a Hg lamp and L5 fluorescence filter (Leica Microsystems, Wetzlar, Germany; 20× objective; 601.7 ms exposure time). At least three images per surface were acquired with a cooled CCD camera (DFC 420C, Leica Microsystems, Wetzlar, Germany). Fluorescence analysis was performed with Fiji software (version 1.53c, Wayne Rasband, National Institute of Health, Bethesda, MD, USA) [41]. Longer incubation times and different microRNA (has-miR-16-Alexa488; miR-16) were also tested.

## 3. Results and Discussion

Silicon surfaces were treated in different plasma conditions with the aim of grafting primary amines in large amounts that were suitable for microRNA capture. Amino-modified surfaces are indeed prone to capturing nucleic acids, but different treatments resulting in differing availability of amines on the surface have been proven to capture different classes of nucleic acids—either DNA or RNA [42,43].

### 3.1. Surfaces Prepared with the Binary Mixture of Gases

#### 3.1.1. Plasma Deposition at Constant Power (200 W)

Silicon surfaces plasma-modified with the binary feed mixture (see Table 1) were firstly characterized via XPS analysis. As shown in Table 2, increasing the relative quantity of NH_3_ in the feed resulted in a decrease in the surface amino nitrogen concentration (as defined in Section 2.3), while the non-amino and the total nitrogen content varied, as previously reported by other groups [44,45].

Moreover, the presence of silicon on the same surfaces was also visible, indicating that the deposited film tends to become thinner or the deposition does not occur when increasing the NH_3_ relative content in the feed. We also observed that the abundance of oxygen correlated with that of silicon, as shown clearly from the SiO_x_ oxidized surfaces of the substrates. At take-off, the angle at 90° of the sampling depth δ was the maximum. Considering that we were analyzing soft amorphous carbon films, δ was estimated to be ~6–8 nm [46]; therefore, the increase in the silicon signal was possibly due to the substrate, which was not completely coated. It should be noted that N was directly bound to C and not to Si, even at the highest percentages of Si shown in Table 2. Here, the BE scale is indeed well aligned and the Si^0^ peak falls at 99 eV, as required. Looking at the N 1s signal no components related to Si–O–NH_2_ are visible, while looking at the Si line no binding with N is present. Therefore, no direct reaction between Si and NH_x_ occurred.

**Figure 1:**
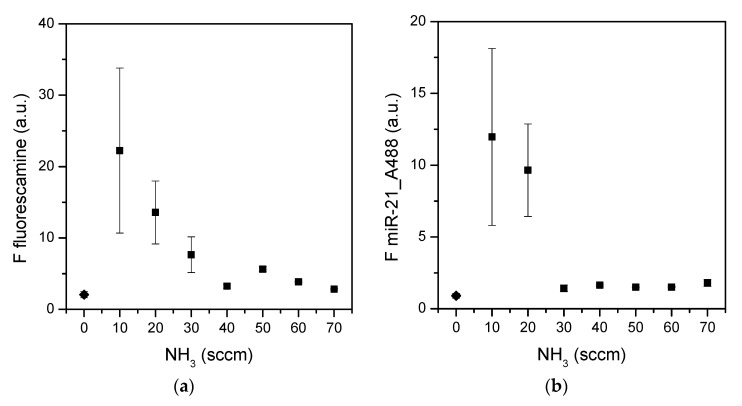
Fluorescamine assay (**a**) and miR-21 adsorption (**b**) on surfaces plasma-processed with CH_4_/NH_3_ feeds. The fluorescence signal (squares) is plotted versus the concentration of NH_3_ in the feed. Values at 0 sccm (diamonds) are control surfaces (untreated silicon). Data are means of at least three surfaces, while the standard error is also presented (*n* ≥ 3).

All surfaces plasma-treated in the binary mixtures were investigated for the accessibility and functionality of the amino groups via fluorescamine assay (see Section 2.3) and microRNA adsorption test (see Section 2.4), respectively (Figure 1).

Both tests were in good agreement with the XPS quantification (Table 2). The highest fluorescence signals were indeed found for surfaces carrying the highest amounts of amino groups. The fluorescamine assay indicated that on such surfaces the amino groups are exposed and accessible for further reactions, while the functional test indicated that miR-21 can be adsorbed on surfaces with more than 3% of amino nitrogen.

Since the surfaces prepared with CH_4_/NH_3_ feeds showed only a slight presence of the desired amino groups, other process conditions were evaluated.

#### 3.1.2. Plasma Deposition at Variable Power

**Figure 2:**
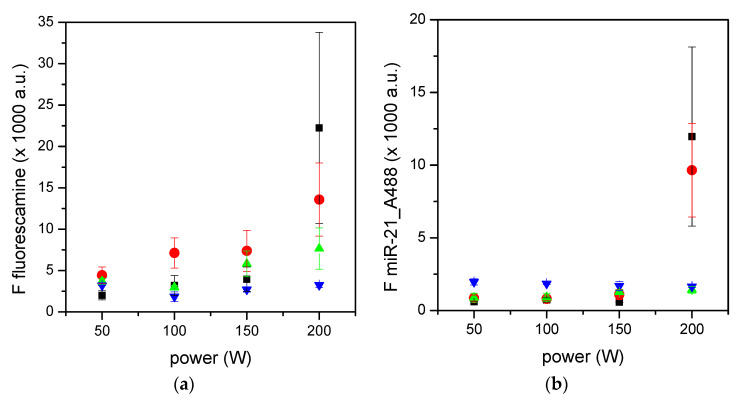
Fluorescamine assay (**a**) and miR-21 adsorption (**b**) on surfaces plasma-coated with binary feeds at varying plasma power levels. Black squares refer to 70:10 CH_4_/NH_3_, red circles refer to 60:20 CH_4_/NH_3_, green upward triangles to 50:30 CH_4_/NH_3_, and blue downward triangles to 40:40 CH_4_/NH_3_. Data are means of at least three surfaces. Standard errors are also reported (*n* ≥ 3).

The analysis of surfaces reported in the previous paragraph revealed that amino groups were present principally on surfaces prepared at lower NH_3_ flow rates. Four conditions, i.e., 10, 20, 30, and 40 sccm of ammonia, were selected to evaluate a possible increase in the deposition of coatings with amino groups by varying the input power. Surfaces were characterized with XPS for the presence of amino groups (Table 3) and with the fluorescence tests for the availability and functionality of these surfaces (Figure 2). The presence of amino groups quantified by XPS was higher on surfaces prepared at the lowest NH_3_ flow rates (i.e., 10 and 20 sccm) and at higher power values, confirming the data obtained at fixed power (Table 2). For the experiments described in the previous paragraph, both the fluorescamine assay and the functional test with miR-21 demonstrated that the best performances were achieved for surfaces with higher amounts of amino groups, i.e., those prepared at 200 W with 10 or 20 sccm of NH_3_.

These experiments allowed us to select 200 W as the optimal power for the plasma deposition process, while a NH_3_ flow rates between 10 and 20 sccm were further evaluated in combination with CH_4_ and H_2_ or N_2_ to obtain more amino groups on silicon surfaces.

### 3.2. Surfaces Prepared with Ternary CH_4_/NH_3_/H_2_ Gas Feeds

**Figure 3:**
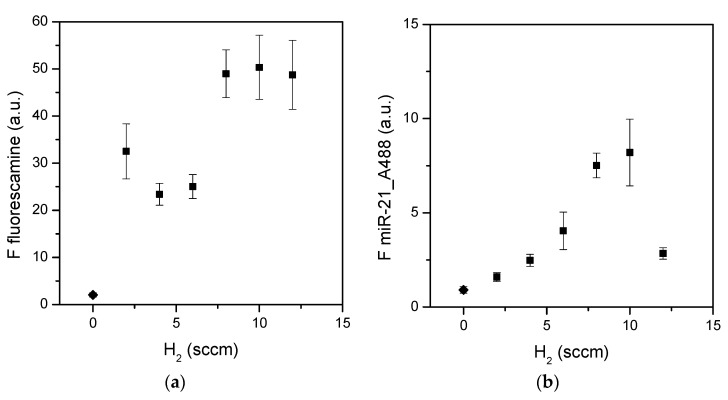
Fluorescamine assay (**a**) and miR-21 adsorption (**b**) on plasma-coated surfaces in the ternary CH_4_/NH_3_/H_2_ gas feed. Fluorescence data (squares) are reported as a function of the H_2_ concentration. Diamonds near zero represent control fluorescence measured on pure silicon surfaces. Data are means of at least three surfaces; standard errors are reported (*n* ≥ 3).

In order to improve the surface functionalization with amino groups, ternary CH_4_/NH_3_/H_2_ gas feeds were tested as precursors in plasma deposition. Six mixtures were used to prepare six different surfaces, which were analyzed with XPS for the presence and quantity of amino nitrogen (Table 4) and characterized with fluorescence assays for the availability and functionality of amino groups (Figure 3). The addition of H_2_ in the feed led to an increment in amino nitrogen on surfaces, even at the lowest (10 sccm) NH_3_ flow rate. Indeed, all ternary mixtures used here resulted in surfaces richer in amino groups with respect to the binary mixtures, as evident when comparing Table 2 with Table 4. Moreover, the percentage of amino nitrogen increased with the increases in NH_3_ and H_2_ concentrations, as expected [45,47].

The amino groups present on the surfaces were perfectly accessible to the fluorescamine molecule, as reported in Figure 3a. An increment in the fluorescence signal was indeed observed for surfaces presenting the highest density of amino nitrogen, not only confirming the increasing presence of primary amines but also a huge fluorescence signal for the three surfaces prepared from the highest NH_3_ and H_2_ contents.

The incremental trend of the fluorescence signal was similarly visible for the functional assay, as shown in Figure 3b. Therefore, the amino nitrogen detected with XPS corresponds to free primary amines, as measured with the fluorescamine assay, and the primary amines contribute to the adsorption of microRNAs molecules. An exception was, however, observed. The surface with the highest amount of primary amines captured only a small amount of miR-21. Moreover, the fluorescence signal due to the capture of miR-21 was not as high as expected from the fluorescamine data for the same surfaces. In other words, the large number of amino groups on surfaces seem not able to capture a similar number of microRNA molecules. To better understand this behavior, other surfaces were prepared starting from a ternary gas feed where N_2_ was used instead of H_2_.

### 3.3. Surfaces Prepared with Ternary CH_4_/NH_3_/N_2_ Gas Feeds

Ternary CH_4_/NH_3_/N_2_ feeds were employed for the preparation of nine types of surfaces via PE-CVD. As with those described in the previous paragraphs, these surfaces were firstly tested for the presence of amino nitrogen by XPS, then for the accessibility of primary amines and for their functionality. Three concentrations of NH_3_, i.e.,10, 15, and 20 sccm, were selected as the most promising for the deposition of coatings with amino groups, while three concentrations of N_2_, i.e., 5, 10, and 15 sccm, were explored for the mixtures (see Table 1). Suitable amounts of CH_4_ were added to obtain a total feed flow rate of 80 sccm (Table 5).

**Figure 4:**
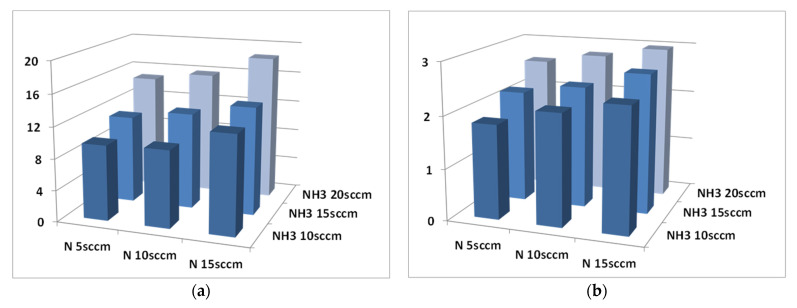
(**a**) Trend of the total N 1s abundance (in %) as a function of the increasing N_2_ and NH_3_ flow rates in the plasma process. (**b**) Trend of the amino-N fraction as a function of the N_2_ and NH_3_ flow rates.

The percentage of amino nitrogen measured by XPS increased with the increase in flow rate of NH_3_ (Table 5 and Figure 4), which was a trend already observed for surfaces prepared with CH_4_/NH_3_/H_2_ mixtures (Table 4). Moreover, when the NH_3_ flow rate was kept fixed, an increase in the amino nitrogen was clearly visible as the concentration of N_2_ increased. The plasma conditions tested in these experiments resulted in coatings with the highest density of amino groups among all those explored in this research. The silicon signal was indeed no more visible in the XPS spectra, attesting to the thicknesses of all coatings being higher than the XPS sampling depth of about 8 nm.

As can be observed in Figure 4a, the total surface concentration of the nitrogen detected on the plasma-treated samples increases by increasing either the flow rate of NH_3_ or that of N_2_ in the plasma process. A similar effect is evident when looking at the abundance of amino groups found on the functional coatings in Figure 4b. This is not surprising because previous studies revealed a linear correlation between the magnitude of the feed flow rate in the PE-CVD reactor and the concentration of the relative functional groups generated [28].

**Figure 5:**
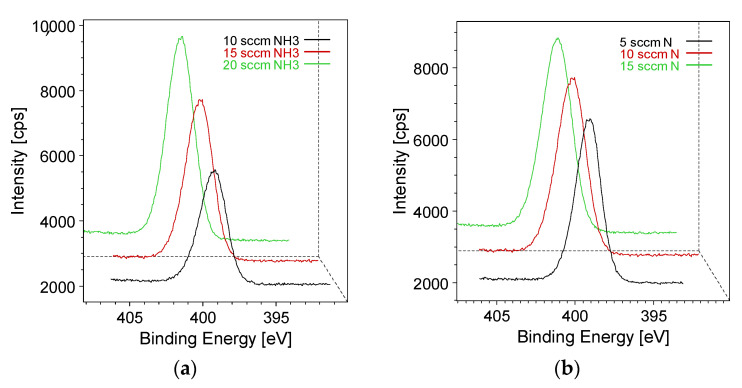
(**a**) Trend of the N 1s intensity as a function of the NH_3_ flow rate with N_2_ constant at 10 sccm. (**b**) Trend of the N 1s intensity as a function of the N_2_ flow rate when keeping the NH_3_ constant at 15 sccm.

This trend is even more evident when looking at the N spectra (Figure 5) both as a function of the NH_3_ flow rate (Figure 5a) and as a function of the N_2_ flow rate (Figure 5b).

**Figure 6:**
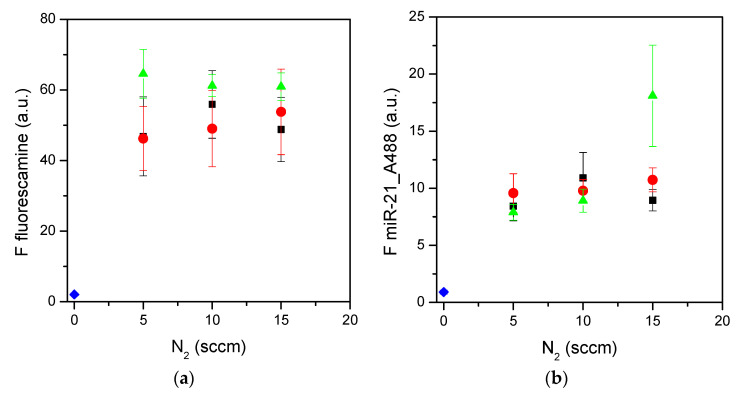
Fluorescamine assay (**a**) and miR-21 adsorption (**b**) on surfaces plasma-coated using CH_4_/NH_3_/H_2_ mixtures. Fluorescence data are reported as a function of the N_2_ flow rate. Black squares refer to 10 sccm NH_3_, red circles to 15 sccm, and green triangles to surfaces prepared with 20 sccm of NH_3_. Blue diamonds near zero represent control fluorescence measured on pure silicon surfaces. Data are means of at least three surfaces; standard errors are reported (*n* ≥ 3).

The amino groups on the surfaces were analyzed for their accessibility and functionality in capturing the microRNA molecules (Figure 6). The general trend of a huge presence of accessible amino groups on surfaces presenting a percentage of amino nitrogen around 6% or higher was confirmed (Figure 6a). In these experimental conditions, the fluorescence signal of fluorescamine reached a plateau for surfaces prepared with either H_2_ or N_2_ as the feed for the plasma. Similarly, the functional assay (Figure 6b) showed good adsorption of miR-21 on all of the surfaces tested, apart from the surface prepared with the 45/20/15 CH_4_/NH_3_/H_2_ feed mixture, which showed the highest adsorption of miR-21. The fluorescence signal measured in the fluorescamine assay was possibly near saturation, on the contrary to the functional assay, where it was still possible to detect a signal increment for the surface with the highest density of amino nitrogen. Therefore, the presence of amino nitrogen nicely correlates with the accessibility of primary amines by small molecules, such as fluorescamine, and to the capture of microRNAs molecules on the same surfaces.

### 3.4. Selection and Analysis of Most Promising Surfaces

The analysis of data presented above points to PE-CVD surfaces prepared with ternary feed mixtures including N_2_. Among these conditions, the 55 sccm CH_4_/10 sccm NH_3_/15 sccm N_2_ feed was selected as an ideal blend to prepare surfaces with good performance in microRNA adsorption. In fact, surfaces prepared in this way showed good percentages of nitrogen, accessibility of amino-charged groups, and capture of miR-21 in line with the plateau performances observed for the best-performing surfaces. These surfaces were, thus, further characterized in terms of the kinetics of microRNAs adsorption, the apparent pH of surface amino groups, and selectivity. The possible elution of microRNAs from these surfaces was also analyzed.

#### 3.4.1. MicroRNA Adsorption as a Function of Time and pH

**Figure 7:**
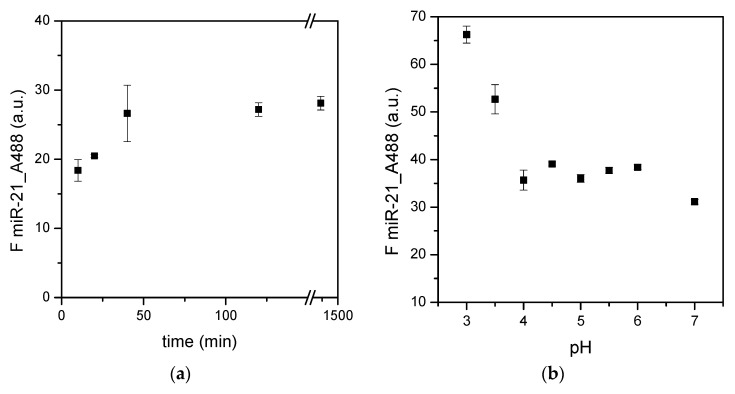
Adsorption at different incubation times of synthetic miR-21 conjugated with the fluorescent dye Alexa488 (**a**) and incubation at different pH levels (**b**). The same amounts of miR-21 were incubated (10 ng). Data are means of at least three surfaces. Standard errors are reported (*n* ≥ 3).

All data concerning microRNA adsorption presented above were collected in standard conditions (i.e., with 20 min of incubation time) and in water (i.e., at pH of or slightly below 7). The time of incubation was further investigated, ranging from no incubation to two hours (Figure 7a). The adsorption of miR-21 increased with time, with a plateau after 30–40 min. An incubation period of 20.5 h was also tested, obtaining a fluorescence signal similar to the plateau, despite the long incubation time. Therefore, an incubation time range of about 20–30 min was confirmed as optimal for this assay, and this was considered a good compromise between the amount of microRNAs captured and the duration of the test. The total duration time of the assay is indeed a parameter to be taken into account, since the final goal of this research was the preparation of surfaces suitable to be included in an analytical test for biomarker analysis.

Another parameter that deserved to be investigated in more detail was the pH of the microRNA incubation. As mentioned above, in all tests presented in this work, synthetic miR-21 was dissolved in water and incubated in an unbuffered environment, which was slightly acidic due to the RNA molecules. Here, the range of pH from 3 to 7 was explored by incubating the same amounts of the fluorescently labeled miR-21 (Figure 7b). The fluorescent dye conjugated to miR-21 is stable in the pH range of 4–10, as declared by the producer [48]. Therefore, the fluorescence levels of known amounts of miR-21_A488 were measured at different pH values with a spectrofluorimeter, in particular at lower acid pH; the results of the experiment reported in Figure 7b were normalized with these values.

The intensity of the fluorescence signal increased at acidic pH, with a maximum value at pH 3. The signal was instead almost stable at pH levels near to the physiological value. Since surface primary amines are protonated depending on the pH, this experiment also allowed us to determine the apparent pK of amine moieties synthesized from the 55/10/15 sccm CH_4_/NH_3_/N_2_ mixture. The apparent pH range of these surfaces was about 3.5–4, as evident from Figure 7b. This value range was similar to that obtained by Vezenov and colleagues [49] both from force microscopy (3.9) and from contact angle wetting (4.3). Interestingly, the surface amine groups in this work were introduced by amino silanes. Similar amino-derived surfaces in contact with RNA molecules gave an apparent pK of about 5.9, when titrated with force vs. distance measurements [50] or with fluorescence [51], while two pKs, 6.5 and 9.9, were measured with a fluorescence-based approach [52]. An apparent pK of around 7 was instead measured for surfaces primary amines introduced with aminothiol molecules [53].

A dependency of microRNA adsorption on the state of ionization (acid–base properties) of amino surface-bound groups was, therefore, measured, with values in good agreement with the literature. As a consequence, the determination of the pK parameter allowed us to set up the conditions for both the adsorption and elution of biomarkers. Circulating biomarkers are indeed dissolved in biological fluids at physiological pH, i.e., around neutral. These conditions should be favorable for microRNAs adsorption, while the release of these molecules should be performed at basic pH.

#### 3.4.2. Selectivity and Elution Performance of Surfaces

Surfaces amino-functionalized with the PE-CVD process fed with the 55/10/15 sccm CH_4_/NH_3_/N_2_ mixture were also tested for the adsorption of miR-16 [54,55], another well-studied microRNA, in order to check whether the adsorption was sequence-specific. MiR-21 and miR-16 have the same length, but obviously different sequences, meaning they could interreact in different ways with the plasma-treated surfaces. To check for this, the two molecules were conjugated with the same dye and tested in parallel conditions.

**Figure 8:**
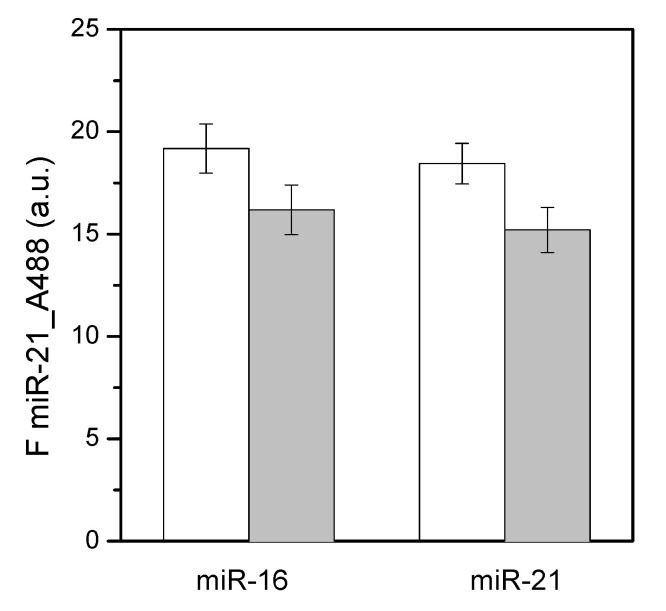
Adsorption (white bars) and elution (gray bars) of synthetic miR-16 or miR-21 fluorescently labeled from surfaces prepared with the 55/10/15 sccm CH_4_/NH_3_/N_2_ mixture. Data are means of at least three surfaces. Error bars refer to standard errors (*n* ≥ 3).

Synthetic miR-16 conjugated with the same fluorescence dye was incubated on the surfaces in the same conditions as miR-21. A similar amount of miR-16 was captured by surfaces (Figure 8), indicating that no specificity for a selected RNA sequence was present, in agreement with previous results [42,51]. Surfaces amino-functionalized by plasma treatments could, therefore, be considered suitable for biomarker microRNA capture.

Beside capture, microRNAs should be released from the amino surfaces to be employed in further analyses. Both miR-16 and miR-21 were eluted from surfaces after a change of the buffer to pH 9. At basic pH, i.e., well above the pK of the surface (Figure 7b), the primary amines are deprotonated (−NH_3_^+^ → −NH_2_) and the electrostatic interactions are minimal. These conditions allow the recovery of microRNAs that are almost complete, i.e., 84.4% for miR-16 and 82.4% for miR-21 (Figure 8). In other words, miR-21 and miR-16 behave in a similar way when tested in similar conditions, allowing us to conclude that our amino-derived surfaces are prone to capture microRNA biomarkers independently from their sequences.

### 3.5. Correlation between microRNA Adsorption and Nitrogen Content

**Figure 9:**
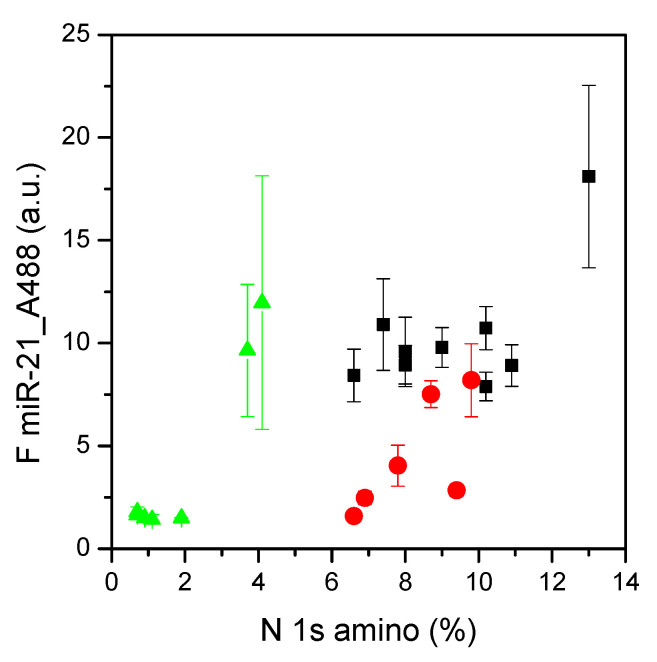
Correlation between miR-21 adsorption and amino nitrogen N 1s XPS components on all surfaces tested. Green triangles refer to surfaces prepared with CH_4_/NH_3_ gas feed, red circles to surfaces prepared with the CH_4_/NH_3_/H_2_ feed, and black squares to surfaces prepared with the CH_4_/NH_3_/N_2_ feed. Data are means of at least three surfaces; standard errors are reported (*n* ≥ 3).

Taking together all data related to miR-21 adsorption on the different plasma-treated surfaces, a correlation with nitrogen content could be observed. An increasing concentration of NH_3_ in the ternary mixtures during the plasma treatment led to an increasing amounts of amino nitrogen (Figure 9), accessible as primary amines to the adsorption of microRNAs. The highest values for both the percentage of amino nitrogen and the adsorption of miR-21 were measured for the surfaces prepared with the highest amounts of NH_3_ and N_2_ (i.e., 45/20/15 sccm of CH_4_/NH_3_/N_2_). This condition, however, showed quite high variability among surfaces tested in parallel experiments, as evidenced by the standard errors shown in Figure 9.

These data indicate that the main parameter for microRNA adsorption is the electrostatic forces between the positively charged amino groups on the surface and the negatively charged backbone of RNA. However, this parameter is not the only one responsible for microRNA adsorption, since other weak forces could be involved, as already reported [16].

As mentioned, some of the results were due to plasma-functionalized surfaces using binary mixtures of gases. In this case, the increment in NH_3_ concentration did not result in an increase in amino nitrogen on the surfaces or in miR-21 adsorption (see triangles in Figure 9). On the contrary, the best adsorption properties were observed for surfaces prepared with 10 or 20 sccm of NH_3_, with a certain degree of variability among the different surfaces, as evident from the standard errors reported in Figure 9. Additionally, these surfaces could have interesting applications in the context of biomarker capture and analysis, suggesting a possible direction for further studies.

## 4. Conclusions

The possibility to capture and purify biomarkers from circulating body fluids is appealing, in particular in the context of rapid and easy-to-use tests and microdevices. Based on our previous experience [17,18,42], a panel of surfaces was prepared via plasma treatments. Binary and ternary gas mixtures were selected as plasma feeds with the aim of functionalizing silicon surfaces with positively charged amino groups, which had already been demonstrated to be capable of capturing microRNA biomarkers [16,42]. The plasma-treated surfaces were tested and a condition belonging to the plateau performances (i.e., ternary gas mixture composed by 55/10/15 sccm of CH_4_/NH_3_/N_2_, see Figure 9) was selected to be further investigated. These surfaces were characterized in terms of the adsorption time and pH and the selectivity and recovery of microRNAs, finding an optimal adsorption time range of 20–30 min, an apparent pK range of 3.5–4, no selectivity for a specific sequence of microRNAs, and an almost complete recovery of microRNAs. These good results certainly need to be confirmed in the field (i.e., with biological samples) and compared to data obtained using the standard liquid biopsy methodologies. However, if these promising results are confirmed, surfaces plasma-coated with the ternary gas feeds could be suggested as good candidates for microdevices such as lab-on-a-chip devices, in particular for the development of miniaturized, portable, low cost, and easy-to use protocols for use in the context of biomarker analyses.

## Figures and Tables

**Table 1:** Binary and ternary mixture of gases used as feeds.

CH_4_ (sccm)	NH_3_ (sccm)	CH_4_ (sccm)	NH_3_ (sccm)	H_2_ (sccm)	CH_4_ (sccm)	NH_3_ (sccm)	N_2_ (sccm)
70	10	68	10	2	65	10	5
60	20	66	10	4	60	10	10
50	30	64	10	6	55	10	15
40	40	57	15	8	60	15	5
30	50	50	20	10	55	15	10
20	60	48	20	12	50	15	15
10	70				55	20	5
					50	20	10
					45	20	15

**Table 2:** XPS chemical compositions (%) of surfaces plasma-processed with CH_4_/NH_3_ feeds (the relative amounts of gases are reported in the first two columns). The definitions for amino, non-amino, and total nitrogen are reported in Section 2.3. PE-CVD was performed at 200 W.

Surface		N 1s			Si 2p	C 1s	O 1s
CH_4_ (sccm)	NH_3_ (sccm)	Amino	Non-Amino	Total			
70	10	4.1	15.5	19.6	0.0	74.7	5.7
60	20	3.9	26.7	30.6	7.5	52.9	8.9
50	30	1.1	13.7	14.8	39.3	15.4	30.5
40	40	0.7	10.0	10.6	40.3	16.6	32.3
30	50	2.0	23.5	25.5	8.6	56.8	9.0
20	60	1.0	9.0	10.0	40.7	18.5	30.7
10	70	2.9	4.9	7.8	45.9	8.4	37.8

**Table 3:** Amino nitrogen (%) values quantified by XPS for surfaces plasma-coated using different relative amounts of CH_4_ and NH_3_ in the feed (reported in the first two columns). The power varied from 50 to 200 W.

Surface		N 1s			
CH_4_ (sccm)	NH_3_ (sccm)	Amino 50 W	Amino 100 W	Amino 150 W	Amino 200 W
70	10	2.6	2.2	3.8	4.3
60	20	2.1	2.3	3.4	3.7
50	30	1.9	1.5	1.1	1.2
40	40	0.7	0.9	0.9	1.0

**Table 4:** Amino nitrogen (%) values quantified by XPS for surfaces plasma-coated with CH_4_/NH_3_/H_2_ gas feeds (reported in columns named “surface”).

Surface			N 1s
CH_4_ (sccm)	NH_3_ (sccm)	H_2_ (sccm)	Amino
68	10	2	6.6
66	10	4	6.9
64	10	6	7.8
57	15	8	8.7
50	20	10	9.8
48	20	12	9.4

**Table 5:** Total and amino nitrogen (%) values quantified by XPS for surfaces plasma-coated in ternary CH_4_/NH_3_/H_2_ mixtures (reported in columns named “surface”).

Surface			N 1 s	
CH_4_ (sccm)	NH_3_ (sccm)	N_2_ (sccm)	Amino	Total
65	10	5	6.6	9.6
60	10	10	7.4	9.8
55	10	15	8.0	12.5
60	15	5	8.0	11.2
55	15	10	9.0	12.3
50	15	15	10.2	13.8
55	20	5	10.2	14.8
50	20	10	10.9	15.8
45	20	15	13.0	18.5

## Data Availability

The data presented in this study are available from the corresponding author upon request.
